# Amorphous‐Like Ultralow Thermal Transport in Crystalline Argyrodite Cu_7_PS_6_

**DOI:** 10.1002/advs.202400258

**Published:** 2024-03-25

**Authors:** Xingchen Shen, Niuchang Ouyang, Yuling Huang, Yung‐Hsiang Tung, Chun‐Chuen Yang, Muhammad Faizan, Nicolas Perez, Ran He, Andrei Sotnikov, Kristin Willa, Chen Wang, Yue Chen, Emmanuel Guilmeau

**Affiliations:** ^1^ Institute for Quantum Materials and Technologies Karlsruhe Institute of Technology 76021 Karlsruhe Germany; ^2^ CRISMAT CNRS ENSICAEN UNICAEN Normandie Univ Caen 14000 France; ^3^ Department of Mechanical Engineering The University of Hong Kong Pokfulam Road Hong Kong SAR China; ^4^ Department of Mechanical and Energy Southern University of Science and Technology (SUSTech) Shenzhen 518055 China; ^5^ Jülich Centre for Neutron Science JCNS at Maier‐Leibnitz Zentrum (MLZ) Forschungszentrum Jülich GmbH Lichtenbergstraße 1 D‐85747 Garching Germany; ^6^ Department of Physics National Central University Chung‐Li District Taoyuan 320317 Taiwan; ^7^ College of Materials Science and Engineering Jilin University Changchun 130012 China; ^8^ Institute for Metallic Materials IFW‐Dresden 01069 Dresden Germany; ^9^ Institute for Solid State Research Leibniz IFW‐Dresden 01069 Dresden Germany

**Keywords:** argyrodite Cu7PS6, crystal structure, Cu Diffusion, lattice dynamics, amorphous‐like ultralow thermal transport

## Abstract

Due to their amorphous‐like ultralow lattice thermal conductivity both below and above the superionic phase transition, crystalline Cu‐ and Ag‐based superionic argyrodites have garnered widespread attention as promising thermoelectric materials. However, despite their intriguing properties, quantifying their lattice thermal conductivities and a comprehensive understanding of the microscopic dynamics that drive these extraordinary properties are still lacking. Here, an integrated experimental and theoretical approach is adopted to reveal the presence of Cu‐dominated low‐energy optical phonons in the Cu‐based argyrodite Cu_7_PS_6_. These phonons yield strong acoustic‐optical phonon scattering through avoided crossing, enabling ultralow lattice thermal conductivity. The Unified Theory of thermal transport is employed to analyze heat conduction and successfully reproduce the experimental amorphous‐like ultralow lattice thermal conductivities, ranging from 0.43 to 0.58 W m^−1^ K^−1^, in the temperature range of 100–400 K. The study reveals that the amorphous‐like ultralow thermal conductivity of Cu_7_PS_6_ stems from a significantly dominant wave‐like conduction mechanism. Moreover, the simulations elucidate the wave‐like thermal transport mainly results from the contribution of Cu‐associated low‐energy overlapping optical phonons. This study highlights the crucial role of low‐energy and overlapping optical modes in facilitating amorphous‐like ultralow thermal transport, providing a thorough understanding of the underlying complex dynamics of argyrodites.

## Graphical abstract

Through experimental and theoretical approaches, it unveils that Cu‐associated low‐energy overlapping optical phonons yield strong phonon–phonon scattering via avoided crossing in argyrodite Cu_7_PS_6_. The domination of wave‐like thermal transport, originating from Cu‐associated low‐energy overlapping optical phonons, also enables its amorphous‐like ultralow thermal conductivity (0.43–0.58 W m^‐1^ K^‐1^) across the temperature range of 100–400 K.

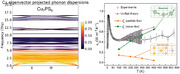

## 1 Introduction

The search for crystalline solids with intrinsically ultralow thermal conductivity holds paramount importance in advancing the development of high‐performance thermoelectric (TE) materials.^[^ ^1^ ^]^ In addition to the extensive studies of conventional solid‐state TE materials, current interest primarily focuses on superionic materials, exemplified by Cu‐ and Ag‐based argyrodites.^[^ ^2^ ^]^ These emerging argyrodites display substantial potential as TE materials owing to their exceptionally ultralow and temperature‐independent lattice thermal conductivity (κ_L_). Typical examples of Cu‐ and Ag‐based argyrodites include Ag_9_GaSe_6_, Ag_8_SnSe_6_, Cu_8_GeSe_6_, and so on, characterized by the general formula A^m+^_(12‐n)/m_ B^n+^X^2−^_6_ (A^m+^ = Cu^+^, Ag^+^, B^n+^ = Al^3+^, Ga^3+^, Si^4+^, Ge^4+^, Sn^4+^, P^5+^, and X^2−^ = S^2−^, Se^2−^, Te^2−^).^[^ ^3^ ^]^ These argyrodites are composed of a large number of atoms in the primitive cell and tend to undergo a phase transition from a low‐temperature ordered phase to a disordered superionic cubic phase upon heating to the critical temperature *T*_c_. In the superionic phase, Cu‐ and Ag‐based argyrodites exhibit a hybrid structure featuring rigid‐mobile sublattices. This structure consists of a rigid framework of BX_4_ tetrahedral, weakly bonded with Cu/Ag ions, along with partially occupied mobile Cu/Ag‐sublattices.^[^ ^3^ ^]^ This disordered mobile Cu/Ag sublattice provides a considerable array of ion configurations with low‐energy barriers, promoting the migration of Cu/Ag ions within the crystal structure. Beyond their similarity in chemical compositions, complex crystal structures, multiple phase transitions, and disordered mobile‐ion sublattice, Cu‐ and Ag‐containing crystalline argyrodites typically exhibit an ultralow and temperature‐independent κ_L_, referred to as amorphous‐like ultralow κ_L_, both below and above the superionic phase transition. Specifically, they manifest an average κ_L_ value of 0.2–0.4 W m^−1^ K^−1^ at 300 K,^[^ ^4^ ^]^ representing the lowest reported value among existing TE materials.

Extensive investigations are currently focused on elucidating the origins of the intrinsically ultralow κ_L_ observed in Cu‐ and Ag‐based argyrodites.^[^ ^2^, ^4^, ^5^ ^]^ The weak bonding force tends to cause a low speed of sound and high phonon‐phonon scattering rates,^[^ ^4^, ^6^ ^]^ which in turn govern ultralow κ_L_. Their complex crystal structures with large unit cells reduce the size of the Brillouin zone, and produce a small portion of heat‐carrying acoustic phonons and a large portion of nondispersive and low‐lying optical phonons, yielding strong acoustic‐optical phonon scattering.^[^ ^7^ ^]^ Moreover, the mobile Cu/Ag‐sublattices exhibit a pronounced interplay between complex lattice dynamics and highly mobile ions across the superionic phase. Recent inelastic neutron scattering (INS) experiments on Cu_7_PSe_6_,^[^ ^8^ ^]^ Ag_8_SnSe_6_,^[^ ^9^ ^]^ and Ag_8_GeSe_6_^[^ ^10^ ^]^ have revealed that the significant Cu‐/Ag‐dominated low‐energy optical phonons were over‐damped and facilitated Cu/Ag diffusions in the superionic phase. Over an extended period, the transverse phonons were anticipated to be suppressed across the superionic phase, and this suppression was expected to achieve ultralow κ_L_.^[^ ^11^ ^]^ However, a recent INS study focusing on a single‐crystal Ag_8_SnSe_6_ sample has shown the persistence of long‐wavelength transverse acoustic phonon across the superionic transition. This observation suggests the likely negligible role of partial melting of Ag sublattices in contributing to the ultralow κ_L_ in the superionic phase.^[^ ^9^ ^]^

Despite some investigations into the microscopic mechanisms underlying the ultralow thermal conductivity and the interplay between soft phonons and mobile ions in crystalline argyrodites, the TE community has initiated studies to understand the origins of amorphous‐like thermal transport. Berngers et al. recently utilized a two‐channel lattice dynamics model to elucidate the observed temperature‐independent κ_L_ of Ag_8_XSe_6_ (X = Si, Ge, and Sn) below the superionic phase transition. They identified that most Ag‐associated vibrations enable diffusive thermal transport,^[^ ^12^ ^]^ indicating their nonpropagating diffuson‐like character. Furthermore, Gupta et al. employed Green–Kubo simulations to reproduce the experimental amorphous‐like κ_L_ of a copper‐based argyrodite Cu_7_PSe_6_ both below and above the superionic phase transition. They identified additional heat contribution channels from phonons, Cu ions, and their cross‐correlations in the superionic phase.^[^ ^8^ ^]^ Notably, the reported crystalline Cu‐/Ag‐based argyrodites^[^ ^4^ ^]^ have already exhibited anomalous amorphous‐like κ_L_ below the superionic phase transitions. However, a comprehensive understanding of the lattice dynamics and their correlations with the underlying amorphous‐like ultralow thermal transports below the superionic phase transition in Cu‐based argyrodites remains scarce.

In this study, we choose Cu_7_PS_6_, a Cu‐based argyrodite, as a showcase material to explore the fundamental relationship between lattice dynamics and amorphous‐like ultralow thermal transport below the superionic phase transition. Our synchrotron X‐ray diffraction (SYXRD) results enable the identification of the superionic phase transition temperature and Cu diffusion channels in Cu_7_PS_6_. Through experiments and theoretical simulations, we revealed the presence of Cu‐dominated low‐energy optical phonons that strongly interact with acoustic phonons via avoided crossing, thereby governing their ultralow thermal conductivity, particularly in the nonsuperionic phase. Importantly, our simulations show a prominently dominant nonpropagating wave‐like phonon transport in Cu_7_PS_6_ and demonstrate that Cu‐associated low‐energy overlapping optical phonons play a crucial role in wave‐like thermal transport in the non‐superionic phase. The computed values of κ_L_, obtained from both the Unified Theory (UT) and nonequilibrium molecular dynamics (NEMD), exhibit excellent agreement with the experimental κ_L_. Thus, our study elucidates the correlations between low‐energy and overlapping optical phonons and amorphous‐like ultralow thermal conductivity in the nonsuperionic phase of Cu‐based argyrodites.

## 2 Results and Discussion

### 2.1 Crystal Structures and Cu Diffusion

**Figure 1:**
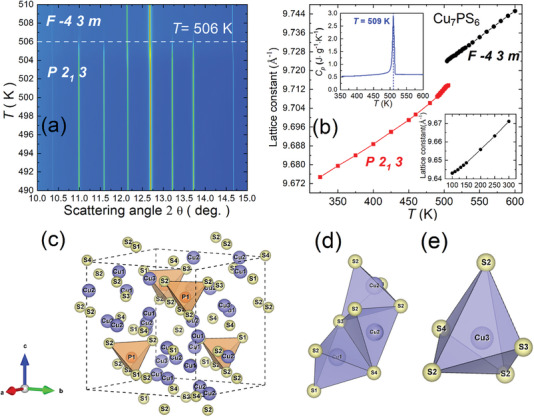
a) Contour plots of the temperature evolution of SYXRD for the Cu_7_PS_6_ powder sample. b) Corresponding temperature‐dependent lattice constant deduced by Rietveld fitting. (Inset) High‐temperature heat capacity measurements of Cu_7_PS_6_ from 350 to 600 K. c) Crystal structure of Cu_7_PS_6_ in a cubic *P*2_1_3 phase at 300 K. Cu positions are marked in purple, and P and S atoms are shown in orange and yellow, respectively. d) four‐coordinated Cu1‐/Cu2‐S tetrahedron and e) five‐coordinated Cu3‐S hexahedron.

The Cu_7_PS_6_ compound has been reported to possess a cubic *P*2_1_3 crystal structure at room temperature and a superionic transition to a cubic *F*‐43m phase at 517 K.^[^ ^13^ ^]^ However, the Cu diffusion channels in the superionic phase remain unexplored. Therefore, we conducted temperature‐dependent SYXRD measurements to validate the crystal structures and, more importantly, to analyze the Cu diffusion pathways in the superionic phase of Cu_7_PS_6_. The contour plot (**Figure** **1**a) and the related refinements of the temperature‐dependent SYXRD (Figure S1, Supporting Information; Figure 1b) revealed the occurrence of the superionic phase transition at 506 K, which is also evidenced by an endothermic peak at ≈509 K in the heat capacity (*C*_p_) measurements (inset of Figure 1b). The identified superionic phase transition temperature and the obtained crystal structures of our sample are consistent with earlier reports.^[^ ^13^, ^14^ ^]^ The low‐temperature cubic *P*2_1_3 phase of Cu_7_PS_6_ (Figure 1c) comprises a framework of PS_4_^3−^ tetrahedron, along with a four‐coordinated Cu‐S tetrahedron (Cu1 and Cu2, Wyckoff 12b) and a five‐coordinated Cu─S hexahedron (Cu3, Wyckoff 4a) (Figure 1d,e; Table S1, Supporting Information). The larger refined isotropic atomic displacement parameters (*U*_iso_) of Cu atoms compared to P atoms (Table S1, Supporting Information) indicate a weak Cu─S bond and a pronounced rattling behavior associated with Cu atoms. This characteristic is likely to facilitate the generation of a relatively higher number of low‐frequency phonons.^[^ ^15^ ^]^

**Figure 2:**
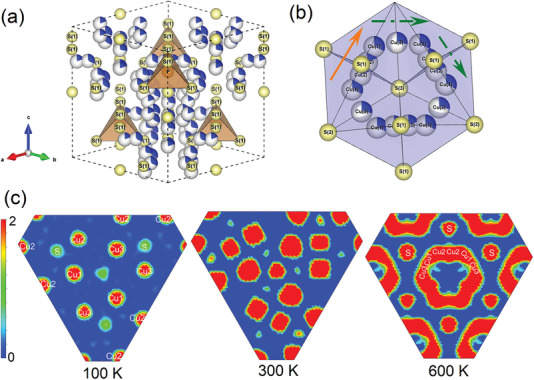
a) Crystal structure of Cu_7_PS_6_ in a cubic *F‐43m* phase at 600 K. Cu positions are displayed with their partial site occupancy (white/blue), and P and S atoms are shown in orange and yellow, respectively. b) Three different sites of Cu1‐Cu3 sublattices with random partial occupation. The solid orange line denotes the direct diffusion channel, while the dashed green line denotes the indirect diffusion pathway, as discussed in the main text. c) 2D electron density maps at 100, 300, and 600 K in (1 1 1) planes.

Elevating the temperature toward the superionic phase leads to a transformation of Cu_7_PS_6_ into the high‐temperature cubic *F*‐43m superionic phase, achieved through a slight rotation of the rigid framework of PS_4_^3−^ tetrahedral along the [111] axis of the non‐superionic cubic *P*2_1_3 phase (**Figure** **2**a). This transition is also accompanied by the presence of three distinct sites within the Cu1‐Cu3 sublattices, characterized by their random partial occupation (Table S2, Supporting Information). In Figure 2b, the Cu atoms occupy two crystallographic positions, specifically, Cu1‐Cu2 atoms of Wyckoff 48 h and Cu3 atoms of 16e (Table S2, Supporting Information). The Cu sublattices with partial occupancy give rise to two distinct diffusion pathways: the Cu1‐Cu2‐Cu2‐Cu1 direct intra‐tetrahedron (marked as the solid orange line in Figure 2b) and the Cu3‐Cu1‐Cu2‐Cu2‐Cu1‐Cu3 indirect inter‐tetrahedron pathway (marked as the dashed green lines in Figure 2b).^[^ ^16^ ^]^ These direct and indirect Cu diffusions through the intra‐ and inter‐tetrahedrons have also been confirmed through molecular dynamics simulation conducted on the analogous Cu‐based argyrodite Cu_7_PSe_6_.^[^ ^8^ ^]^

To explicitly visualize the Cu diffusion pathways of Cu_7_PS_6_ in the superionic phase, we deduced the temperature‐dependent 2D electron density maps (EDMs) through Rietveld refinements of SYXRD data (Figure 2c). The EDMs along the (111) plane at 100 and 300 K distinctly exhibit the separately well‐defined electron spots of Cu1‐Cu3 atoms in the low‐temperature cubic *P*2_1_3 phase. However, upon heating to the high‐temperature cubic *F*‐43m superionic phase, the electron spots of Cu atoms become interconnected (e.g., at 600 K), signifying the occurrence of diffusion of Cu atoms in Cu_7_PS_6_. For a quantitative assessment of Cu diffusion, we calculated the temperature‐dependent mean square displacement (MSD) of Cu, P, and S atoms, as illustrated in Figure S2a–c (Supporting Information). The MSD values of P and S atoms remain relatively constant below the superionic phase transition, such as at 300 K (Figure S2a, Supporting Information) and 500 K (Figure S2b, Supporting Information). In contrast, the MSD values of Cu atoms display a linear increase in the superionic phase, e.g., at 550 K (Figure S2c, Supporting Information), confirming its Cu diffusion characteristics. The linear fit of MSDs against time (*t*) yields a diffusion coefficient *D* of 5.5 × 10^5^ cm^2^ s^−1^ at 550 K [(Figure S2c, Supporting Information), see the method in the experimental section for further details].

### 2.2 Lattice Dynamics

**Figure 3:**
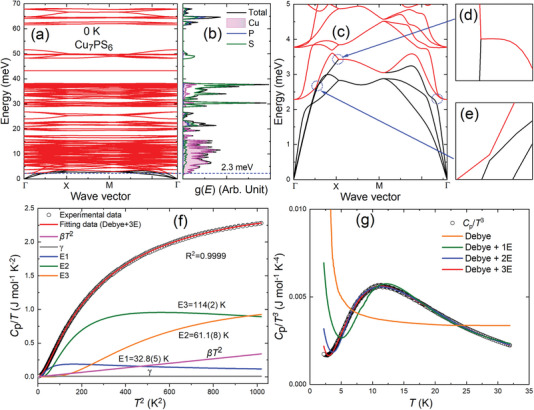
The calculated a) phonon dispersion and corresponding b) PDOS for Cu_7_PS_6_ at 0 K. The blue dashed lines denote the low‐energy optical phonon modes. The c–e) magnified phonon dispersion for Cu_7_PS_6_ at 0 K. The avoided crossing points are marked in dashed blue circles. f) The fitting profile of the experimental data for *C*_p_/*T* versus *T* ^2^ with Debye and three Einstein terms. g) The fitting profiles of the temperature dependence of *C*_p_/*T^3^* plot.

To unveil the details of the lattice dynamics of Cu_7_PS_6_, we use a combination of experimental and theoretical methods. As depicted in **Figure** **3**a, the calculated phonon dispersion at 0 K features low‐energy optical modes of 2.3–3.6 meV (blue dashed line in Figure 3a), along with numerous overlapping optical phonons above 2.3 meV due to the large number of atoms per unit cell (*N *= 56). In this context, optical phonons that overlap within the range of 2.3–15 meV are referred to as low‐energy overlapping optical phonons. These low‐energy overlapping optical phonons are primarily dominated by Cu‐associated vibrations (atom‐resolved phonon dispersion in Figure S3, Supporting Information and pink shaded regions in the phonon density of states (PDOS) in Figure 3b), while the intermediate and high‐energy phonon modes above 20 meV are mainly contributed by P and S atoms. In Figure 3c, the magnified phonon dispersion shows the lowest frequency of optical phonons at 2.3 meV, and some prominent avoided crossing points (denoted as blue dashed circles in Figure 3c–e) between longitudinal acoustic (LA) phonons and low‐energy optical phonons of 2.3–3.6 meV, which indicates the emergence of strong phonon–phonon coupling in Cu_7_PS_6_.

Such avoided crossing feature results in the softening of the LA mode, a decrease in LA mode group velocity, and an increase in LA scattering rates, which enables the ultralowκ_L_.^[^ ^17^ ^]^ We further computed the three‐phonon and four‐phonon scattering rates of Cu_7_PS_6_ and found the dominant role of three‐phonon interactions in Cu_7_PS_6_ over the whole phonon frequency regime, as shown in Figure S4 (Supporting Information). The calculated three‐/four‐ phonon scattering rates exhibit high values in the frequency region of the coupling between LA and low‐energy optical phonons, ranging from 0.6–1.1 THZ (equivalent to 2.5–4.5 meV, denoted as black dashed lines in Figure S4, Supporting Information). This validates that the avoided crossing drives giant anharmonic acoustic‐optical phonon interactions, resulting in a high scattering rate.

To experimentally confirm the existence of low‐energy vibrations, we conducted measurements of low‐temperature *C*_p_ and depicted the data as *C*_p_/*T* versus *T* ^2^ in the temperature range of 2–32 K (Figure 3f). In addition to the lattice contribution of the single Debye model, we further analyze the low‐temperature experimental data by incorporating three additional Einstein modes. By applying the Debye–Einstein model, expressed in Equation (1), we successfully fitted our experimental data, as indicated by the solid red line in Figure 3f.

(1) $$\frac{C_{p}}{T}=\;\gamma +\beta T^{2}+\sum_{n}\left(A_{n}{\left(\Theta_{E_{n}}\right)}^{2}\cdot {\left(T^{2}\right)}^{-3/2}\cdot \frac{e^{\Theta_{E_{n}}/T}}{{\left(e^{\Theta_{E_{n}}/T}-1\right)}^{2}}\right)$$

where 𝛾 represents the Sommerfeld coefficient, which denotes the electronic contribution to *C*_p_, while the term *β* describes the lattice contribution based on the Debye model. Moreover, the Debye temperature (*Θ*_D_) can be determined from the fitting parameter β using Equation (2):

(2) $$\beta \;=\;C\left(\frac{12\pi^{4}N_{A}k_{B}}{5}\right)\cdot {\left(\Theta_{D}\right)}^{-3}$$

where *N*_A_ and *k*_B_ represent Avogadro's number and Boltzmann constant, respectively. The parameter *C* can be estimated from $C\;=\;1-\sum_{n}A_{n}/3\mathrm{NR}$, where *N* and *R* are the numbers of atoms per formula unit and the universal gas constant, respectively. In Equation (1), the third term denotes the lattice contribution from the independent Einstein oscillators, namely E1, E2, and E3. *A*_n_ and $\Theta_{E_{n}}$ represent the variable coefficients and Einstein temperatures of the nth Einstein mode, respectively. Using the Debye–Einstein model, we obtain $\Theta_{D}$ ***Θ***_**D**_ = 155, $\Theta_{E1}$ = 32.8(5), $\Theta_{E_{2}}$ = 61.1(8), and $\Theta_{E_{3}}$ = 114(2) K with the other fitting parameters listed in Table S3 (Supporting Information). In Figure 3g, the invalid fitting profiles of *C*_p_/*T* ^3^‐*T* plot with Debye and one/two Einstein terms, especially in the low‐temperature range of 2–15 K, further verify the reliable fitting of heat capacity through the addition of three Einstein terms. Moreover, we observed an obvious broad peak, referred to as the Boson peak,^[^ ^2h^ ^]^ between 10 and 15 K in the *C*_p_/*T* ^3^‐*T*

plot, which might arise from the collective behavior of low‐energy vibrational excitations or the interactions between these excitations.^[^ ^2h^ ^]^ Hence, based on the heat capacity fitting and analysis, we identified the low‐energy optical phonon modes characterized as $\Theta_{E_{1}}$ (= 2.8 meV), in agreement with the calculated low‐energy optical phonons of 2.3–3.6 meV (Figure 3a). The phonon energies of other Einstein modes (E2 = 5.3 meV, E3 = 9.8 meV) reasonably fall within the calculated low‐energy overlapping optical phonon energy regime of ≈2.3–15 meV.

We also determined the fitting average sound velocity ν_a, fit_ at the base temperature with a low value of ≈1312 m ^−1^s from the estimated value of *Θ*_D_ (Table S3, Supporting Information). The estimated value of ν_a, fit_ is roughly in agreement with the calculated average sound velocity ν_a, cal_ at 0 K (Table S4, Supporting Information). The ν_a, cal_ increases from ≈1750 m ^−1^s at 0 K to ≈2327 m ^−1^s at 300 K, and its value at 300 K is consistent with the measured average sound velocity ν_a, exp_ values of 2395 m ^−1^s (Table S5, Supporting Information) at 300 K. The origins of the elevated sound velocities as temperatures are beyond the scope of the present study. Furthermore, the substantial difference between the measured longitudinal and transverse sound velocities at 300 K results in high Gru ¨neisen parameter γ(1.99) and Poisson ratio ν_p_(0.33) in Cu_7_PS_6_ (Table S5, Supporting Information). These derived large values are comparable to that of other Cu‐/Ag‐ based argyrodites,^[^ ^4^ ^]^reflecting its intrinsically soft lattice and strong anharmonicity.^[^ ^18^ ^]^

**Figure 4:**
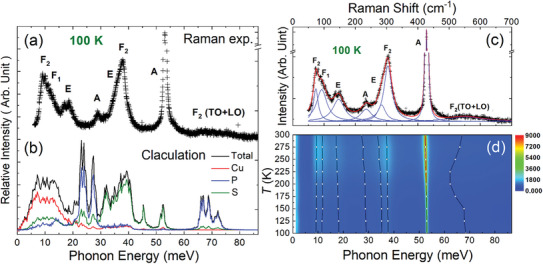
a) Raman spectrum and b) the calculated PDOS for Cu_7_PS_6_ at 100 K. The phonon energy of the calculated PDOS is multiplied by a factor of 1.06 for comparison. c) The fitting profile of the Raman spectrum at 100 K. The black cross symbols represent experimental values; the blue solid line illustrates the fitted peaks, while the red solid line indicates the sum of the fitted peaks. d) The contour temperature‐dependent Raman spectrum is from 100 to 300 K, where hollow circles mark the energy positions of the fitted Raman peaks.

To assess the temperature evolution of the optical phonons, we performed Raman spectroscopy measurements on the Cu_7_PS_6_ sample from 100 to 300 K (Figure S5, Supporting Information, see the Experimental Section for details). **Figure** **4**a displays the Raman spectrum of the Cu_7_PS_6_ sample at 100 K, encompassing Raman shifts from 50 to 700 cm^−1^. The Raman peaks at 74 cm^−1^ (F_2_, triple) and 92 cm^−1^ (F_1_, triple) arise from the diffusive‐type vibrations of Cu atoms,^[^ ^19^ ^]^ and the Raman peak located at 146 cm^−1^ (E) and 235 cm^−1^ (A) originate from the out‐of‐phase breathing mode of Cu and S sublattices.^[^ ^19a^ ^]^ The Raman peaks at 284 cm^−1^ (E), 304 cm^−1^ (F_2_, triple), and 428 cm^−1^ (A) correspond to the bending vibrations and symmetric stretching vibrations of the PS_4_ tetrahedral.^[^ ^19^, ^20^ ^]^ Within the range of 500–600 cm^−1^, a broad and relatively weak Raman peak is observed, which stems from the transverse optical and longitudinal optical vibrations of the F_2_ mode.^[^ ^19^, ^20^ ^]^ Moreover, we compare the Raman peaks (Figure 4a) with the calculated PDOS at 100 K (Figure 4b), and observe a notable consistency between the theoretical vibrational modes and experimentally observed Raman active modes.

In Figure 4c, we fitted the full‐profile Raman spectrum with eight Raman peaks using the Lorentzian functions. The obtained Cu‐dominated low‐energy overlapping phonons of the Raman peaks, specifically F_2_@74 cm^−1^ (equivalent to 9.2 meV), F_1_@92 cm^−1^ (equivalent to 11.4 meV) (black solid spheres in Figure S6, Supporting Information), exhibit reasonable agreement with the simulated data near the Gamma point in the Brillouin zone of the calculated phonon dispersion at 100 K (Figure S6, Supporting Information). As the temperature increases, it is important to note that the other six Raman peaks, including those above 146 cm^−1^, exhibit a softening trend (Figure 4d) and broadening phenomena (full width at half maximum (FWHM), as shown in Figure S7, Supporting Information), which collectively indicate the presence of anharmonic vibrational characteristics at elevated temperatures. In contrast, the Cu atomic diffusive‐type Raman peaks at 74 and 92 cm^−1^ do not follow this trend. They even show a slightly hardening Raman shift and a narrower half‐peak width with increasing temperature, implying that some Cu atoms might become more actively engaged in pre‐diffusion within Cu's sublattice as the temperature rises.

### 2.3 Amorphous‐Like Ultralow Lattice Thermal Conductivities

**Figure 5:**
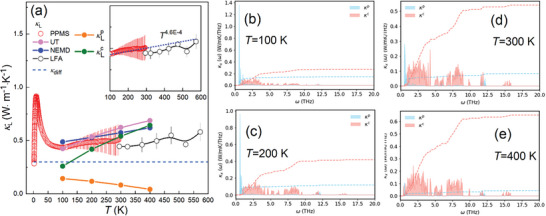
a) The experimental lattice thermal conductivities for Cu_7_PS_6_ from 2 to 573 K. The simulated κ_L_ based on the UT and NEMD are shown from 100 to 400 K. The inset figure displays magnified experimental κ_L_ ranging from 100 to 573 K. The simulated spectral lattice thermal conductivity ($\kappa_{L}^{p}$ and $\kappa_{L}^{c}$) at c) 100 K, d) 200 K, e) 300 K, and f) 400 K.

We measured the temperature‐dependent κ_L_ of Cu_7_PS_6_ from 2 to 573 K [for detailed methodology, refer to the experimental section]. The Cu_7_PS_6_ sample displays exceptionally ultralow lattice thermal conductivities, ranging from ≈0.28 to 0.58 W m^−1^ K^−1^ (**Figure** **5**a). The measured values are above the minimal value estimated from diffusive thermal conductivity, κ_diff_ of ≈0.30 W m^−1^K^−1^ using ν_a, cal_ of 1750 m ^−1^s^[^ ^21^ ^]^ (blue dashed line in Figure 5a). Notably, it features an Umklapp crystalline peak of 0.9 W m^−1^ K^−1^ at an exceptionally low temperature of ≈10 K. Generally, under the approximation of an acoustic‐phonon dominated scattering mechanism, the temperature‐dependent κ_L_ in crystals with simple structures follows an inverse power law of κ_L_∝*T* ^−1^.^[^ ^22^ ^]^ Nonetheless, the temperature‐dependent κ_L_ of the Cu_7_PS_6_ sample follows a power law of κ_L_∝*T* ^4.6*E* − 4^ from 100 to 573 K (inset of Figure 5a), approaching the amorphous limit κ_L_∝*T* ^0^.^[^ ^22^ ^]^ Therefore, despite its crystalline structure, the Cu_7_PS_6_ sample exhibits an amorphous‐like ultralow K_L_ in the temperature range of 100–573 K, evoking similarities with the thermal transport behavior observed in amorphous materials.^[^ ^23^ ^]^

In accordance with the heat conduction mechanisms proposed by Allen and Feldman for the amorphous or disordered systems,^[^ ^24^ ^]^ the short‐range heat carriers with phonon mean paths either shorter than or comparable to the Ioffe–Regel limit undergo wave‐like diffusion through a Zener‐like tunneling between quasi‐degenerate vibrational eigenstates. While in crystals with complicated structures, i.e., Cu_7_PS_6_, due to the substantial number of atoms per unit cell and the smaller minimum q wave‐vector in reciprocal space, a multitude of optical phonon modes overlap within the crystal structure. Since the majority of these phonon modes possess comparable frequencies, exhibit low phonon group velocities, and engage in frequent collisions, the stored heat can efficiently transfer among those nonpropagated phonon branches through wave‐like tunneling (analogous to Zener‐like tunneling in amorphous systems).^[^ ^25^ ^]^ This contributes to an additional nonpropagating wave‐like heat conduction channel, apart from the contribution of the propagating particle‐like phonons using the phonon‐gas model within the framework of the Boltzmann transport equation.^[^ ^26^ ^]^

Considering the complexity and the significant overlapping optical phonons in Cu_7_PS_6_, the following quantitative assessment of thermal conductivity for the Cu_7_PS_6_ sample will consider the contribution of wave‐like heat conduction. Based on the recently developed UT in complex crystals, which integrates wave‐like and particle‐like phonon transports in Wigner functions,^[^ ^26^ ^]^ we have applied this framework to quantitatively analyze the thermal transport in the temperature range of 100–400 K for Cu_7_PS_6_. It is important to note that within the superionic phase, complexities arise due to the accurate description of heat conduction in overdamped phonons,^[^ ^25^ ^]^ convection heat transfer involving mobile Cu ions, and complex scattering interactions between vibrational phonons and mobile Cu ions, especially in complex materials. Hence, the quantitative assessment of temperature‐dependent lattice thermal conductivity for Cu_7_PS_6_ in the superionic phase (above 500 K) falls beyond the scope of the current work.

According to the scheme proposed by Simoncelli et al.,^[^ ^26^ ^]^ the K_L_ comprises heat contributions from the conventional particle‐like term ($\kappa_{L}^{p}$) and wave‐like term ($\kappa_{L}^{c}$), as described by the following equation:^[^ ^26^ ^]^

(3) $$\kappa_{L}=\kappa_{L}^{p}\;+\kappa_{L}^{c}$$

The equation for κ_L_ can be expressed as a square matrix, wherein the decoupled diagonal term and off‐diagonal term represent the particle‐like and wave‐like components, respectively.^[^ ^26^ ^]^ In anharmonic complex crystals, the interband spacing of nonpropagating wave‐like phonons is typically much smaller than the phonon line widths. As a result, the highly anharmonic nonpropagating phonon bands not only exhibit couplings between nearly degenerate vibrational modes but also show strong couplings among phonon modes with markedly different frequencies at elevated temperatures.^[^ ^25^ ^]^ Consequently, besides the heat contribution from nonpropagating phonons with comparable frequencies, stored heat can transfer among various nonpropagating wave‐like phonon branches spanning a wide frequency range, leading to an increased thermal conductivity as temperatures rise.^[^ ^25^ ^]^

As illustrated in Figure 5a, the calculated κ_L_ values and trends derived from the UT exhibit reasonable agreement with experimental data, falling within the acceptable error bars from 100 to 400 K. Moreover, we conducted κ_L_ calculations using NEMD simulations (see method details in the Experimental Section), accounting for full‐order lattice anharmonicity. The resultant values also exhibit agreement with our experimental findings. To analyze the evolution of phonon frequency in $\kappa_{L}^{p}$ and $\kappa_{L}^{c}$, we further extracted the spectral K_L_ from 100 to 400 K (Figure 5b–e). At 100 K, $\kappa_{L}^{p}$ is mostly dominated by acoustic phonons within the range of ≈0.6–1.0 THZ (2.5–4.1 meV), while $\kappa_{L}^{c}$ is mainly comprised of the low‐frequency overlapping optical phonons in the range of ≈0.6–3.7 THZ (2.5–15.0 meV) (Figure 5b).

The overall κ_L_ consists of a 35.6% (0.144 W m^−1^ K^−1^) contribution from the particle‐like term and a 64.4% (0.260 W m^−1^ K^−1^) contribution from the wave‐like term at 100 K, signifying the prominence of its wave‐like thermal transport characteristics. Upon increasing temperatures to 400 K, the strong anharmonicity progressively diminishes $\kappa_{L}^{p}$ from 0.144 (35%) to 0.044 (6.3%) W m^−1^ K^−1^. Contrarily, $\kappa_{L}^{c}$ undergoes a notable increase from 0.270 (65%) to 0.654 (93.7%) W m^−1^ K^−1^. Furthermore, the low‐energy overlapping optical phonons within the 0.6–3.7 THZ range constitute 77% of $\kappa_{L}^{c}$ at 100 K, suggesting their dominance contribution to wave‐like thermal transport of Cu_7_PS_6_. The proportion of contribution for $\kappa_{L}^{c}$ from the overlapping phonons above 3.7 THZ increases from 23% to 45% between 100 and 400 K. These increments are facilitated by enhanced net heat diffusion between phonon frequencies that vary significantly, driven by the presence of strong anharmonicity at elevated temperatures.^[^ ^25^ ^]^ The spectral κ_L_ demonstrates that the amorphous‐like thermal transport in Cu_7_PS_6_ originates from its prominently dominant wave‐like thermal transport. Our simulations also verify the considerable role played by the Cu‐associated low‐energy overlapping optical phonons in enabling this amorphous‐like thermal conductivity.

## 3 Conclusion

In summary, our study explored the correlations between lattice dynamics and amorphous‐like ultralow thermal transport through a comprehensive combination of experimental and theoretical methods. We successfully identified the superionic phase transition at ≈506 K and directly visualized the Cu diffusion channel via SYXRD. In the high‐temperature superionic phase, Cu ions exhibit a remarkably large diffusion coefficient of 5.5 × 10^−5^ cm^2^ s^−1^ at 550 K through MSD analysis. Notably, the low‐energy optical phonons exhibit a strong anharmonic interaction with LA phonons through avoided crossing. We quantitatively examined the temperature evolution of K_L_ from 100 to 400 K in the framework of UT and NEMD. We found that significant dominant wave‐like thermal transport in facilitating amorphous‐like thermal conductivity in the non‐superionic phase of Cu_7_PS_6_. In a nutshell, our study provides an in‐depth investigation of the microscopic mechanisms governing the amorphous‐like ultralow K_L_ in the nonsuperionic phase of Cu‐based argyrodites. It demonstrates that Cu‐associated low‐energy and overlapping optical phonon transport dominate amorphous‐like ultralow thermal conductivity in Cu_7_PS_6_. We hope that our findings offer valuable insights to guide the rational manipulation of low‐energy overlapping optical phonons, thereby enabling controlled modulation of thermal transport in argyrodites for high TE performance.

## 4 Experimental Section

### Materials Synthesis

The polycrystalline Cu_7_PS_6_ sample with an amount of 3 g was synthesized using melting‐annealing and hot‐pressing (HP) synthetic routes. Stoichiometric amounts of high‐purity Cu (Shot, 99.99%), P (Shot, 99.999%), and S (Block, 99.99%) elements were loaded, sealed, and evacuated to a vacuum of ≈10^−4^ Pa inside a silica tube. The silica tube was slowly heated to 723 K for 10 h, held at this temperature for 2 h, and then heated to 1333 K for 10 h and maintained at this temperature for an additional 24 h. The resulting ingot was ground into fine powder by hand and further subjected to vacuum annealing at 973 K for a duration of 30 h. Subsequently, the annealed sample was densified by HP at 45 MPa for 30 min. This HP treatment results in a highly dense sample with a density exceeding 97% of the theoretical density.

### Structure Characterization

Temperature‐dependent synchrotron X‐ray diffraction data were acquired from a powder sample of ≈1 mg, utilizing a wavelength of 0.7749 Å. The measurements were conducted from 100 to 600 K, employing the TPS09A beamline at the National Synchrotron Radiation Research Center (NSRRC), Taiwan Photon Source. The analysis of all synchrotron X‐ray diffraction data was performed using the Rietveld method and the GSAS (General Structure Analysis System).^[^ ^27^ ^]^

### Thermal Conductivity and Heat Capacity Measurements

The high‐temperature overall thermal conductivity (κ_tot_) was determined using the formula κ_tot_ =  ρ*C*_p_ *d*, where *d* represents the thermal diffusivity, which was measured utilizing a Netzsch LFA 457 laser flash system under a nitrogen atmosphere in the temperature range of 300 to 573 K. The isobaric heat capacity (*C*_p_) was measured employing a Differential Scanning Calorimeter (Netzsch 404 F3) from 350 to 600 K, and the density *ρ* was determined via the Archimedes method. Here, it used the values of overall thermal conductivity for experimental values of κ_L_ as the contribution of electronic thermal conductivity to κ_tot_ was found to be negligible. The low‐temperature heat capacity and thermal conductivity were obtained using a Quantum Design Physical Property Measurement System in the temperature range of 2 to 300 K.

### Sound Velocity Measurements

The measurements were carried out by a RITEC Advanced Ultrasonic Measurement System RAM‐5000 at room temperature. The system employed the ultrasonic pulse‐echo technique of time propagation measurements. To generate and receive longitudinal (L) and shear (S) ultrasonic bulk waves, Olympus transducers V208‐RM (20 MHz) and V157‐RM (5 MHz) were utilized, respectively. Propylene glycol and SWC (both from Olympus) were used as couplant materials for L and S modes, respectively. The achieved time resolution in the experiments surpassed 0.2 ns. Thickness measurements were performed using the Mitutoyo ID‐HO530 device. Figure S8 (Supporting Information) displays the pulse‐echo pattern (first and second echoes) for the L mode.

### Raman Experiment

Raman spectra were collected from 100 to 300 K using HORIBA Jobin Yvon's modular systems, equipped with an iHR 550 spectrometer and a 632.8 nm laser. A Linkam THMS600 microscope stage was employed to control the sample temperature.

### Density Functional Theory and Molecular Dynamics Simulations

Nonspin polarized density functional theory (DFT) calculations for Cu_7_PS_6_ were performed using the Vienna Ab initio Simulation Package (VASP)^[^ ^28^ ^]^ with the projector augmented wave (PAW)^[^ ^29^ ^]^ method. The PBEsol^[^ ^30^ ^]^ exchange‐correlation functional was applied throughout the calculations. The self‐consistent electronic calculations employed a plane wave energy cutoff of 500 eV and an energy convergence criterion of 10^−8^ eV. A Monkhorst–Pack *k*‐mesh^[^ ^31^ ^]^ of 1 × 1 × 1 was utilized to sample the Brillouin zone of the 448‐atom supercell of Cu_7_PS_6_. The optimized lattice parameters at 0 K were used for the subsequent calculations. To simulate the partial occupancy of Cu atoms in the high‐temperature cubic phase of Cu_7_PS_6_ (Figure S9, Supporting Information), the general special quasi‐random structure (SQS) algorithm ^[^ ^32^ ^]^ implemented in USPEX was employed.^[^ ^33^ ^]^ Specifically, seven Cu atoms were mixed with 21 vacancies (corresponding to 28 Cu partial occupation lattice sites).

The moment tensor potential (MTP) of Cu_7_PS_6_ was constructed using the machine‐learning interatomic potentials software package.^[^ ^34^ ^]^ The distorted reference configurations of Cu_7_PS_6_ comprise 230 single‐point DFT calculations, which were generated from ab initio molecular dynamics (AIMD) simulations with a 448‐atoms supercell at elevated temperatures up to 400 K under the *NVT* ensemble. The 10% of the configurations that were not included in the training were randomly selected to validate the accuracy and transferability of the MTP, as shown in Figure S10 (Supporting Information). The MSD was computed using the formula:

(4) $$MSD\;\left(t\right)=\;\frac{\sum_{i\;=\;1}^{N}{\left(r_{i}\left(t\right)-r_{i}\left(t_{0}\right)\right)}^{2}}{N}$$

In this equation, *N* represents the number of atoms, and *r_i_* (*t*) is the position of atom *i* at time *t*. The diffusion coefficient (*D*) of ions can then be calculated from their MSD using the Einstein–Smoluchowski equation, given by:

(5) $$D\;=\frac{MSD}{6\times t}\;$$

Here, *t* denotes the time interval.

The temperature‐dependent renormalized second‐order force constants were extracted using the temperature‐dependent effective potential (TDEP) method, following the work of Hellman et al.,^[^ ^35^ ^]^ as implemented in the hiPhive package.^[^ ^36^ ^]^ Figure S11 (Supporting Information) shows the phonon dispersions of Cu_7_PS_6_ calculated using the TDEP method at different temperatures. AIMD simulations were performed over a temperature range of 100 to 400 K, and 50 configurations were randomly extracted to fit temperature‐dependent cubic and quartic force constants. The neighboring cutoff distances for pairs, triplets, and quadruplets of Cu_7_PS_6_ were set to 7.5, 6.0, and 3.5 Å, respectively. Besides, the κ_L_(*L*) of Cu_7_PS_6_ with a finite length *L* is also calculated based on nonequilibrium molecular dynamics simulations using Fourier's law: $\kappa_{L}\;\left(L\right)=\;\frac{Q}{S|\frac{\nabla T}{\nabla L}|},$ where *Q* is the heat flux, *S* is the cross‐sectional area perpendicular to the thermal transport direction, and |*∇T*| is the temperature gradient. Four independent simulations were performed for each system with different lengths *L* to determine the asymptotic values of κ_L_. The obtained results are presented in Figure S12 (Supporting Information).

## Conflict of Interest

The authors declare no conflict of interest.

## Author Contributions

X.S., N.O., and Y.H. contributed equally to this work. X.S. conceived the idea and designed the project. X.S., Y.H., and E.M. performed materials synthesis and sintering. Y.H., N.P., and R.H. performed thermal transport properties measurements. Y.H. and K.W. performed heat capacity measurements. A.S. sound velocity measurements. C.‐C.Y. performed Raman spectroscopy measurements. Y.‐H.T. and C.‐C.Y. performed synchrotron X‐ray diffraction experiments and refinements. N.O., C.W., and Y.C. performed calculations. X.S.; C‐C.Y. performed data analysis. X.S, C.‐C.Y., N.O., and Y.C. performed the manuscript draft. X.S., M.F., C.‐C.Y., Y.C., and E. M. performed Review and editing.

## Supporting information

Supporting Information

## Data Availability

The data that support the findings of this study are available from the corresponding author upon reasonable request.
