# Supplementary material for: Amorphous‐Like Ultralow Thermal Transport in Crystalline Argyrodite Cu7PS6
Source: Adv Sci (Weinh). 2024 Mar 25;11(22):2400258. doi: 10.1002/advs.202400258 (PMC11165551; doi:10.1002/advs.202400258)
Supplement: Supplementary file 1 — Supporting Information [file ADVS-11-2400258-s001.pdf]

ADVANCED SCIENCE Open Access

Supporting Information

for *Adv. Sci.*, DOI 10.1002/advs.202400258

Amorphous-Like Ultralow Thermal Transport in Crystalline Argyrodite Cu_7PS_6

Xingchen Shen, Niuchang Ouyang, Yuling Huang, Yung-Hsiang Tung, Chun-Chuen Yang*, Muhammad Faizan, Nicolas Perez, Ran He, Andrei Sotnikov, Kristin Willa, Chen Wang, Yue Chen* and Emmanuel Guilmeau*

Supplemental information

Amorphous-like Ultralow Thermal Transport in Crystalline Argyrodite Cu_7PS_6

Xingchen Shen[1, 2, #,*], Niuchang Ouyang[3, #], Yuling Huang[4, #], Yung-Hsiang Tung[5, 6], Chun-Chuen Yang[6,*], Muhammad Faizan[7], Nicolas Perez[8], Ran He[8], Andrei Sotnikov[9], Kristin Willa[1], Chen Wang[3], Yue Chen[3,*], Emmanuel Guilmeau[2]

[1]Institute for Quantum Materials and Technologies, Karlsruhe Institute of Technology, 76021 Karlsruhe, Germany
[2]CRISMAT, CNRS, Normandie Univ, ENSICAEN, UNICAEN, 14000 Caen, France
[3]Department of Mechanical Engineering, The University of Hong Kong, Pokfulam Road, Hong Kong SAR, China
[4]Department of Mechanical and Energy, Southern University of Science and Technology (SUSTech), Shenzhen 518055, China
[5]Jülich Centre for Neutron Science JCNS at Maier-Leibnitz Zentrum (MLZ), Forschungszentrum Jülich GmbH Lichtenbergstraße 1, D-85747 Garching, Germany
[6]Department of Physics, National Central University, Chung-Li District, Taoyuan City, 320317, Taiwan
[7]College of Materials Science and Engineering, Jilin University, Changchun 130012, China
[8]Institute for Metallic Materials, IFW-Dresden, Dresden, 01069, Germany
[9]Institute for Solid State Research, Leibniz IFW Dresden, Dresden, 01069, Germany

[#] These authors contributed equally to this work
* Authors to whom correspondence should be addressed:
Xingchen Shen: xingchen.shen@ensicaen.fr
Chun-Chuen Yang: chunchuenyang@ncu.edu.tw
Yue Chen: yuechen@hku.hk

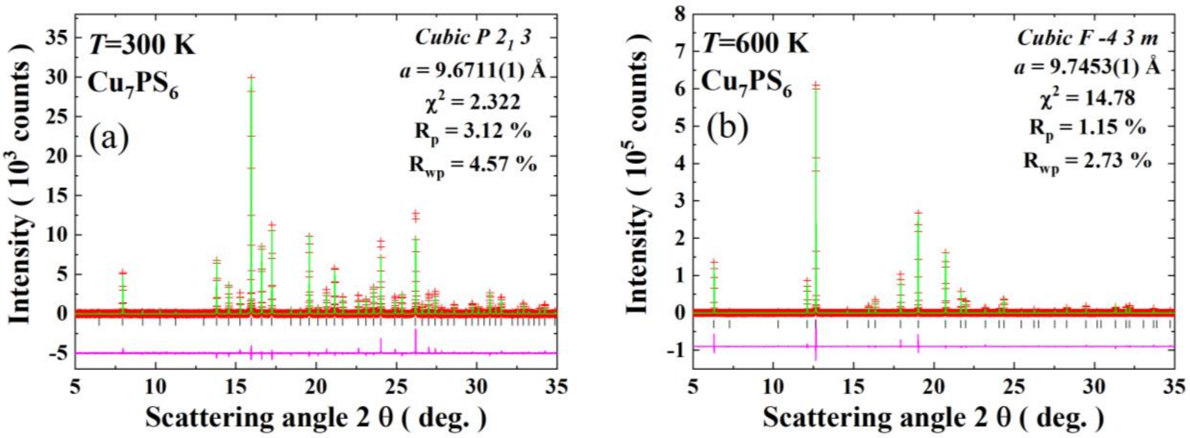

Figure S1. Rietveld refinements of the Cu_7PS_6 powder sample at representative temperature of (a) 300 and (b) 600 K.

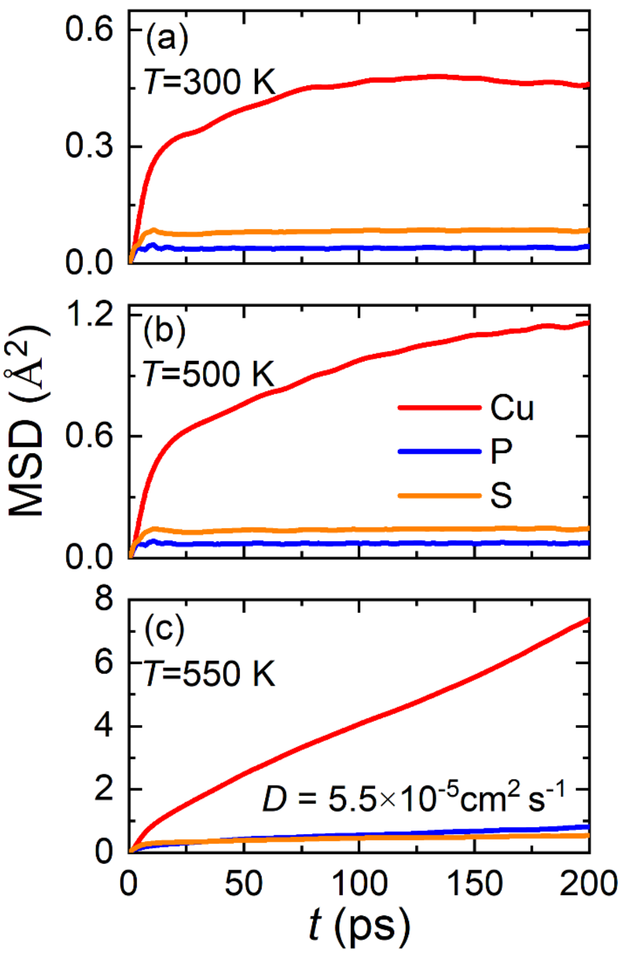

Figure S2. The calculated atomic mean-square displacement (MSD) at (a) 300 K, (b) 500 K, and (c) 550 K. The red, blue, and orange solid lines denote Cu, P, and S atoms, respectively.

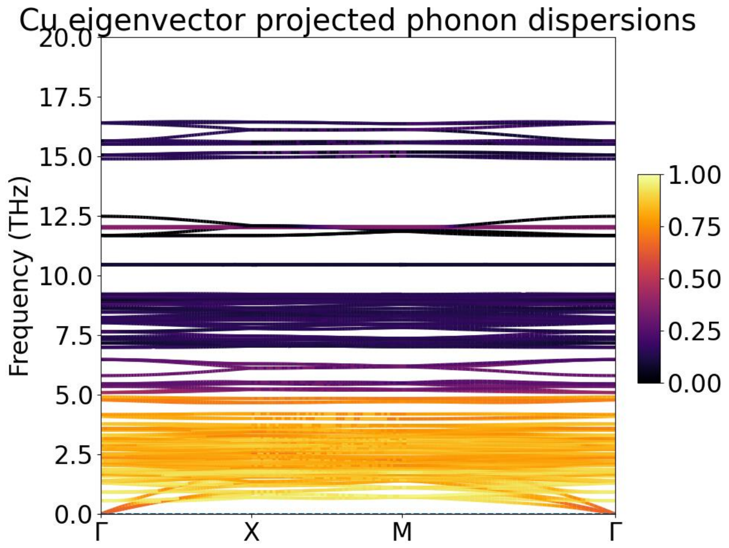

Figure S3. Simulated harmonic phonon dispersion of Cu_7PS_6. The colours refer to the module square of Cu components of the phonon eigenvector. As eigenvectors of each mode are normalized to one, the maximum of colourbar value is one.

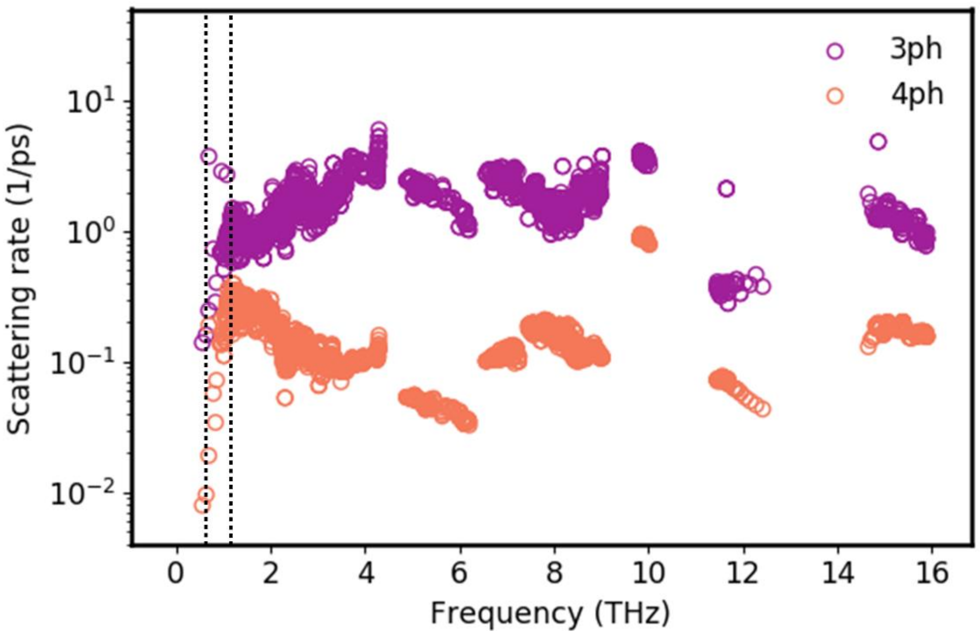

Figure S4. The computed scattering rate of Cu_7PS_6 with three-phonon and four-phonon scattering.

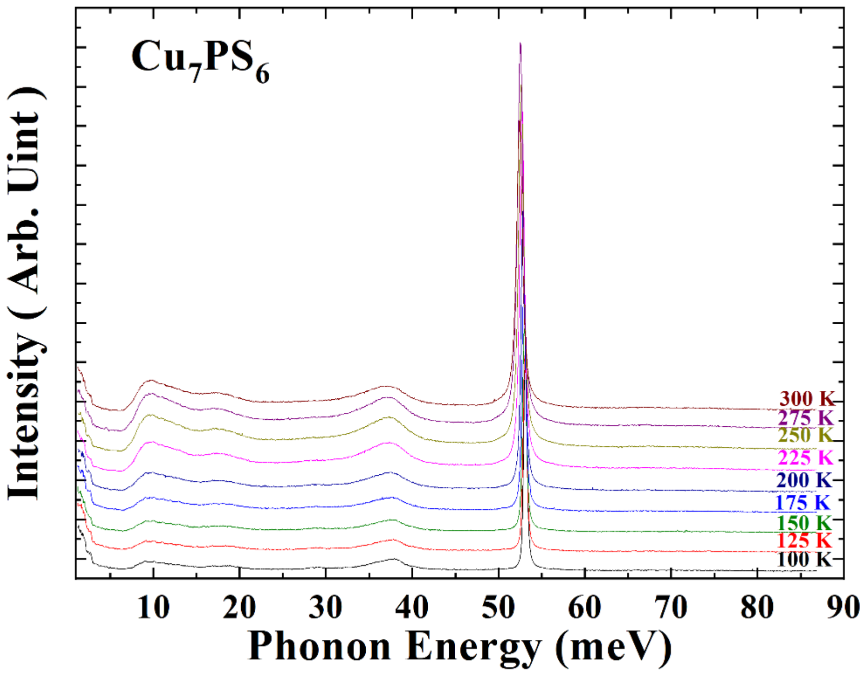

Figure S5. Raman spectrum of the Cu_7PS_6 sample from 100 to 300 K.

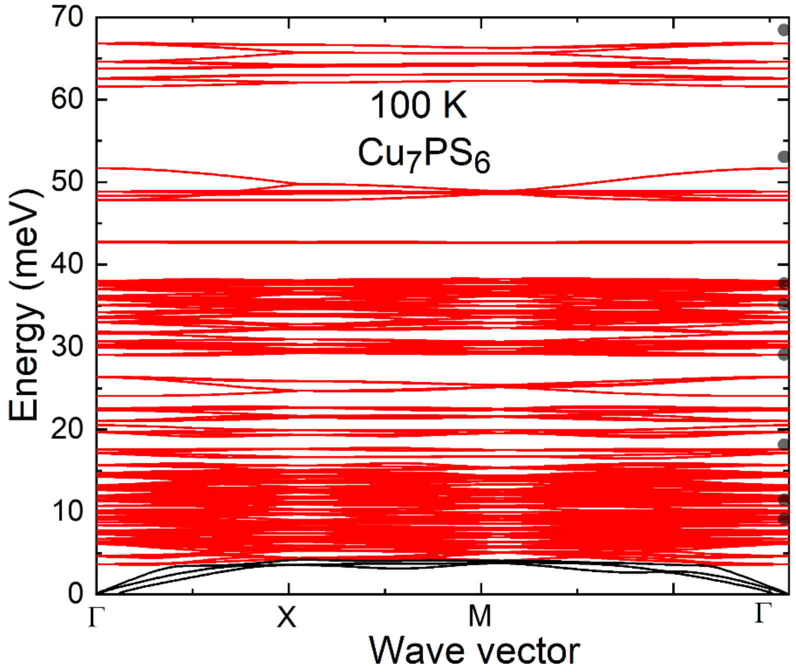

Figure S6. The calculated phonon dispersion for Cu_7PS_6 at 100 K. The black solid spheres represent the derived optical phonons from Raman spectrum at 100 K.

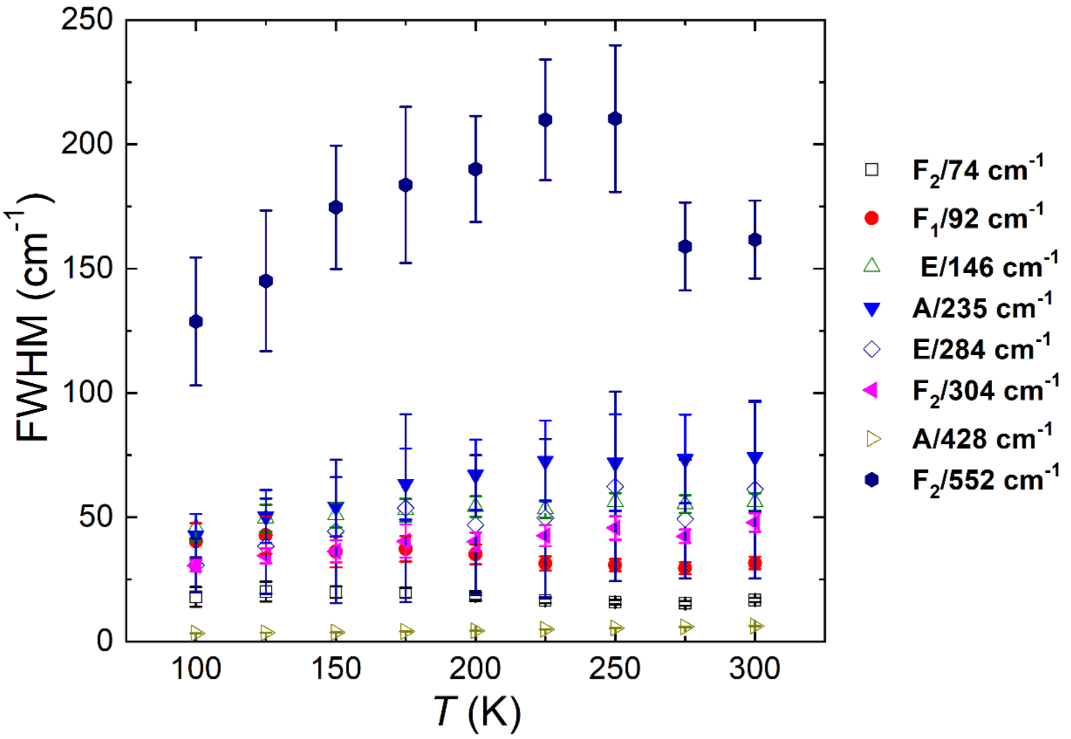

Figure S7. The full width at half-maximum (FWHM) derived from the eight fitted Raman peaks for the Cu_7PS_6 sample.

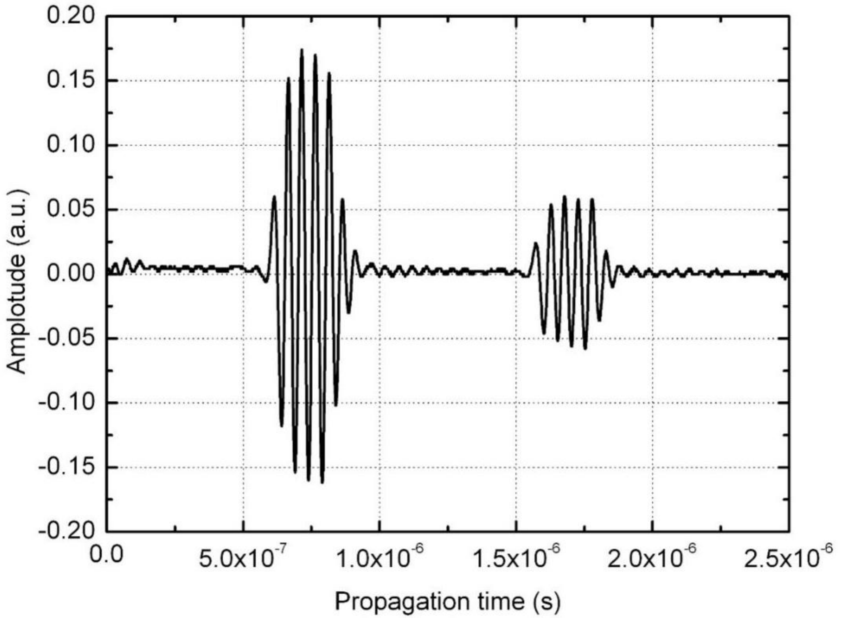

Figure S8. Pulse-echo pattern (frequency 20 MHz) for the longitudinal mode.

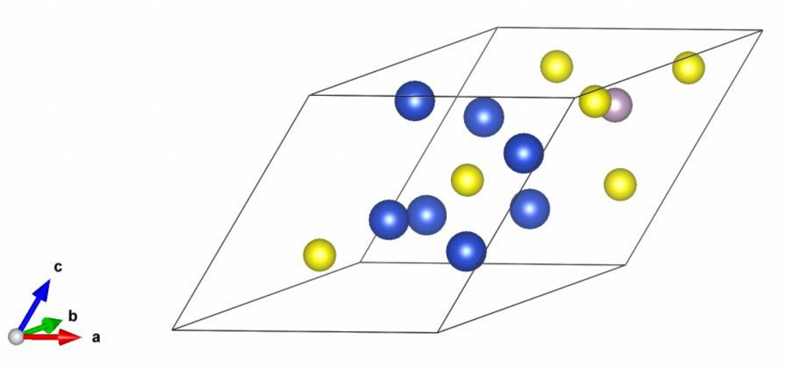

Figure S9. Crystal structure of the high-temperature phase of Cu_7PS_6 rendered using VESTA.[1] Cu, P, and S atoms are shown in blue, purple, and yellow, respectively.

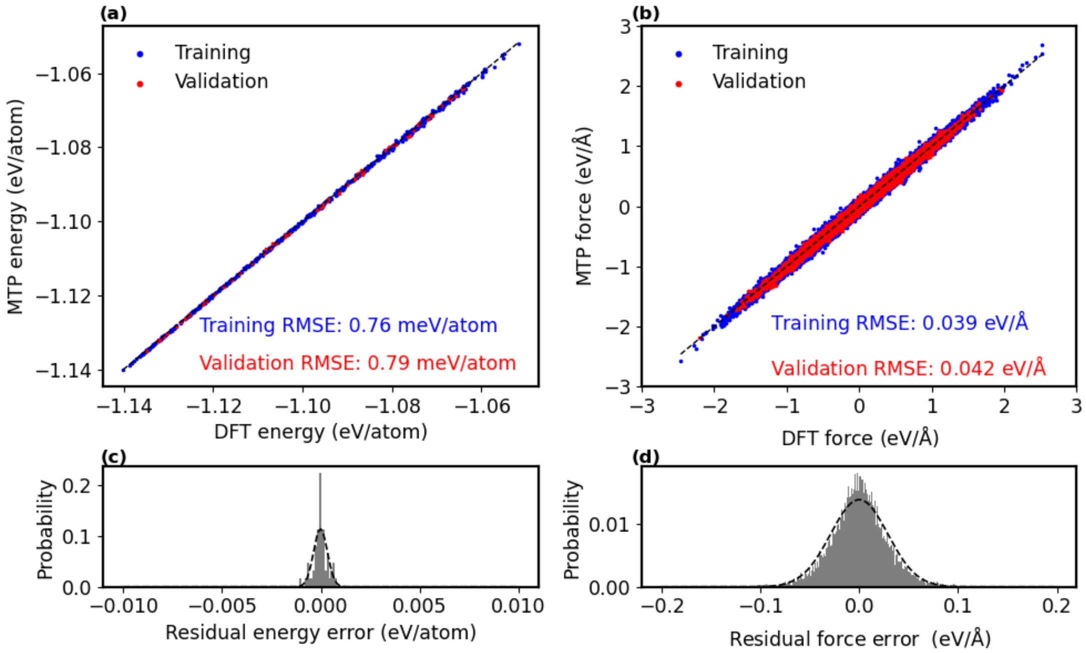

Figure S10. The root mean square errors (RMSEs) of (a) energies and (b) atomic forces of Cu_7PS_6 in the training and validation sets. The distribution of the MTP residual errors for (c) energies and atomic (d) forces in the validation set. A normal distribution fitted to the residual errors for the validation set is shown as a black dashed curve.

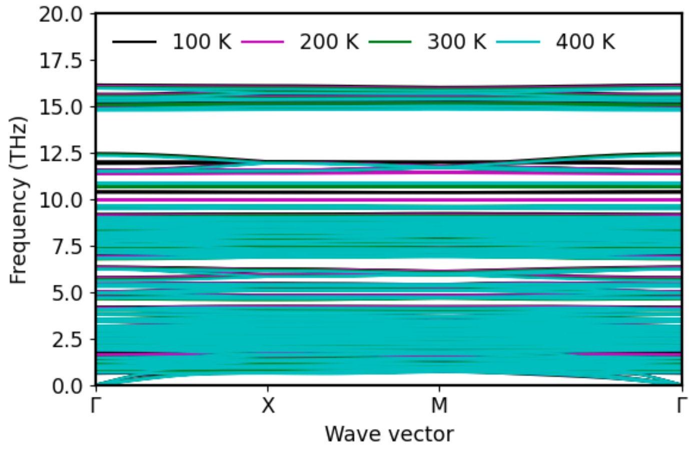

Figure S11. Renormalized phonon dispersions of Cu_7PS_6 calculated using the TDEP method at different temperatures.

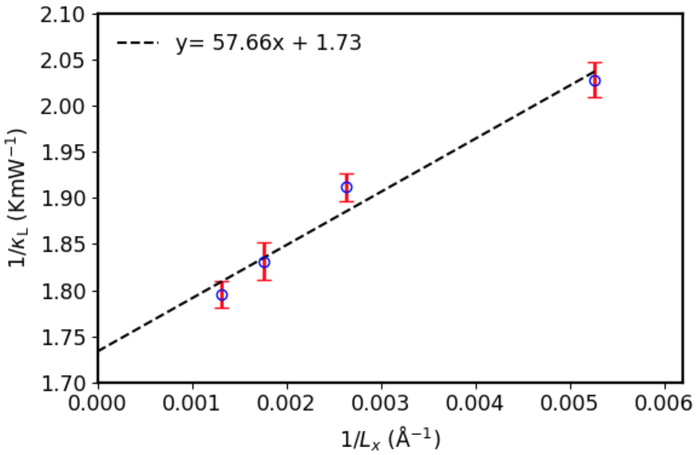

Figure S12. System size dependence of $1/\kappa_{\mathrm{L}}$ on $1/L_x$ for Cu_7PS_6 at 300 K. Dashed line is a linear fitting of the Matthiessen's rule: $\frac{1}{\kappa_{\mathrm{L}}(L)} = \frac{1}{k_\infty}(1 + \frac{4l_\infty}{L_x})$, where κ_∞ is the bulk lattice thermal conductivity and l_∞ is the mean free path in an infinite system.

Table S1. Atomic refined parameters of cubic phase $P2_13$ of Cu_7PS_6 at 300 K.

Atomic parameters						
Atom	Wyck.	x	y	z	Occ.	U_{iso}
Cu1	12b	0.0736	0.4071	0.2592	1.000	0.045
Cu2	12b	0.2258	0.4878	0.4839	1.000	0.046
Cu3	4a	0.1366	0.1366	0.1366	1.000	0.080
P	4a	0.7529	0.7529	0.7529	1.000	0.005
S	4a	0.6261	0.6261	0.6261	1.000	0.013

Table S2. Atomic refined parameters of cubic phase F-43m of Cu_7PS_6 at 600 K.

Atomic parameters						
Atom	Wyck.	x	y	z	Occ.	U_{iso}
Cu1	48h	0.0811	0.7500	0.2564	0.315	0.078
Cu2	48h	0.0274	0.8741	0.2738	0.175	0.034
Cu3	16e	0.1297	0.1297	0.3703	0.271	0.214
P	4d	0.7500	0.7500	0.7500	1.000	0.031
S1	16e	0.8741	0.8741	0.8741	1.000	0.023
S2	4c	0.2500	0.2500	0.2500	1.000	0.017
S3	4b	0.5000	0.5000	0.5000	1.000	0.035

Table S3. Fitting parameters of low-temperature C_p of Cu_7PS_6.

Composition	γ (J mol^{-1} K^{-2})	β (×10^{-4} J mol^{-1} K^{-4})	Θ_D (K)	A1 (J mol^{-1} K^{-1})	Θ_{E1} (K)	A2 (J mol^{-1} K^{-1})	Θ_{E2} (K)	A3 (J mol^{-1} K^{-1})	Θ_{E3} (K)
Cu_7PS_6	0.009(5)	3.34(2)	155	4.1(3)	32.8(5)	38(1)	61.1(8)	77(2)	114(2)

Table S4. The calculated sound velocities obtained from the slopes of the linear segments of the acoustic branches along the Γ-X and Γ-M directions in the calculated phonon dispersion for Cu_7PS_6.

T	Γ-X			Γ-M		
	v_{LA} (m s^{-1})	v_{TA} (m s^{-1})	$v_{a,cal}$ (m s^{-1})	v_{LA} (m s^{-1})	v_{TA} (m s^{-1})	$v_{a,cal}$ (m s^{-1})
0 K	3045	1605	1795	2910	1535	1716
100 K	3460	1975	2195	3380	1945	2160
200 K	3625	2080	2310	3585	2005	2232
300 K	3740	2160	2372	3780	2045	2282

Table S5. Experimental sound velocity and elastic properties of Cu_7PS_6 at room temperature.

Composition	v_l (m s^{-1})	v_t (m s^{-1})	$v_{a,exp}$ (m s^{-1})	v_p	E (GPa)	G (GPa)	Υ
Cu_7PS_6	4257	2135	2395	0.33	59.5	12.8	1.99

Supplemental Note 1:

Calculations of average sound velocity and elastic parameters

The average sound velocity and elastic parameters of Cu_7PS_6 were calculated from the measured longitudinal sound velocity (v_l) and transverse sound velocity (v_t) using the following formulas:[2]

$$v_{\mathrm{a,exp}} = \left[\frac{1}{3}\left(\frac{1}{v_{\mathrm{l}}^3} + \frac{2}{v_{\mathrm{t}}^3}\right)\right]^{-\frac{1}{3}} \qquad \text{S3}$$

$$v_{\mathrm{p}} = \frac{1-2(\frac{v_{\mathrm{t}}}{v_{\mathrm{l}}})^2}{2-2(\frac{v_{\mathrm{t}}}{v_{\mathrm{l}}})^2} \qquad \text{S4}$$

$$E = \frac{\rho v_{\mathrm{t}}^2(3v_{\mathrm{l}}^2-4v_{\mathrm{t}}^2)}{v_{\mathrm{l}}^2-v_{\mathrm{t}}^2} \qquad \text{S5}$$

$$G = \frac{E}{2(2+v_{\mathrm{p}})} \qquad \text{S6}$$

$$\gamma = \frac{3}{2}\left(\frac{1+v_{\mathrm{p}}}{2-3v_{\mathrm{p}}}\right) \qquad \text{S7}$$

Where $v_{\mathrm{a,exp}}$, v_{p}, *E*, *G*, and γ are the average sound velocity, Poisson ratio, Young's modulus, shear modulus, and Grüneisen parameter, respectively.

References

[1] K. Momma, F. Izumi, *Journal of Applied crystallography* **2008**, 41, 653.

[2] a)S. Lin, W. Li, Y. Pei, *Materials Today* **2021**, 48, 198; b)B. Jiang, P. Qiu, H. Chen, Q. Zhang, K. Zhao, D. Ren, X. Shi, L. Chen, *Chemical Communications* **2017**, 53, 11658; c)B. Jiang, P. Qiu, E. Eikeland, H. Chen, Q. Song, D. Ren, T. Zhang, J. Yang, B. B. Iversen, X. Shi, L. Chen, *Journal of Materials Chemistry C* **2017**, 5, 943; d)X. Shen, C.-C. Yang, Y. Liu, G. Wang, H. Tan, Y.-H. Tung, G. Wang, X. Lu, J. He, X. Zhou, *ACS Applied Materials & Interfaces* **2019**, 11, 2168; e)Y. Tang, Y. Yu, N. Zhao, K. Liu, H. Chen, C. C. Stoumpos, Y. Shi, S. Chen, L. Yu, J. Wu, Q. Zhang, X. Su, X. Tang, *Angewandte Chemie International Edition* **2022**, 61, e202208281.
